# The crystal structure of XdpB, the bacterial old yellow enzyme, in an FMN-free form

**DOI:** 10.1371/journal.pone.0195299

**Published:** 2018-04-09

**Authors:** Jiří Zahradník, Petr Kolenko, Andrea Palyzová, Jiří Černý, Lucie Kolářová, Eva Kyslíková, Helena Marešová, Michal Grulich, Jaroslav Nunvar, Miroslav Šulc, Pavel Kyslík, Bohdan Schneider

**Affiliations:** 1 Institute of Biotechnology CAS, v. v. i., BIOCEV, Vestec, Prague West, Czech Republic; 2 Department of Biochemistry, Faculty of Science, Charles University Prague, Czech Republic; 3 Institute of Microbiology CAS, v. v. i., Prague, Czech Republic; 4 Dept. of Solid State Engineering, FNSPE Czech Technical University, Prague, Czech Republic; NCI at Frederick, UNITED STATES

## Abstract

Old Yellow Enzymes (OYEs) are NAD(P)H dehydrogenases of not fully resolved physiological roles that are widespread among bacteria, plants, and fungi and have a great potential for biotechnological applications. We determined the apo form crystal structure of a member of the OYE class, glycerol trinitrate reductase XdpB, from *Agrobacterium bohemicum* R89-1 at 2.1 Å resolution. In agreement with the structures of the related bacterial OYEs, the structure revealed the TIM barrel fold with an N-terminal β-hairpin lid, but surprisingly, the structure did not contain its cofactor FMN. Its putative binding site was occupied by a pentapeptide TTSDN from the C-terminus of a symmetry related molecule. Biochemical experiments confirmed a specific concentration-dependent oligomerization and a low FMN content. The blocking of the FMN binding site can exist *in vivo* and regulates enzyme activity. Our bioinformatic analysis indicated that a similar self-inhibition could be expected in more OYEs which we designated as subgroup OYE C1. This subgroup is widespread among G-bacteria and can be recognized by the conserved sequence GxxDYP in proximity of the C termini. In proteobacteria, the C1 subgroup OYEs are typically coded in one operon with short-chain dehydrogenase. This operon is controlled by the tetR-like transcriptional regulator. OYEs coded in these operons are unlikely to be involved in the oxidative stress response as the other known members of the OYE family because no upregulation of XdpB was observed after exposing *A*. *bohemicum* R89-1 to oxidative stress.

## Introduction

Members of the Old Yellow Enzyme family (OYEs) are NAD(P)H dehydrogenases containing noncovalently bound flavin-mononucleotide (FMN). These enzymes have been reported in bacteria, yeasts, fungi, plants, invertebrates, and rarely also in higher animals. OYEs 3D structures adopt the highly conserved (αβ)_8_ TIM-barrel fold with a closed, central β-barrel. A characteristic feature of OYEs is the β-hairpin lid formed by the N-terminal amino acids and a well conserved FMN binding site formed by the C-terminal edges of the β-barrel. The enzyme catalysis is based on His/His or His/Asn pairs of residues. The catalytic pair acts as an H-bond donor to the substrate electron-withdrawing group (e.g. carbonyl group) [1–5]

The first OYE was isolated by Warburg in 1932 as the first flavoprotein [6]. OYEs are involved in a host of functions. OYEs are known to have physiologic substrates such as 12-oxophytodienoate involved in jasmonate biosynthesis in plants [7], they degrade metabolic products of oxidative stress after lipid peroxidation [8], play a role in yeast apoptotic processes [9], or they take part in the reductive degradation of xenobiotics. Differential expression of OYE genes during oxidative stress and plant infection shows their involvement in multiple pathways within one organism [10].

Especially OYEs´ ability to degrade xenobiotics opens new ways for applications in medicine and biotechnology. For instance, their ene-reductase activity allows an effective, asymmetric hydrogenation of α, β-unsaturated double bonds with a high selectivity and reaction yield [11], their nitro-reductase activity is useful for bioremediation applications [12]. Involvement of OYEs in xenobiotics degradation suggests they can be promising drug targets. In parasite *Trypanosoma cruzi*, which causes Chaga´s disease, TcOYE catalyzes the reduction of Nifurtimox and other trypanocidal drugs [13]. Another example of OYE catalysis is a single-electron reduction of the drug menadione by TcOYE [14], or catabolism of acrolein by OYE2 of *S*. *cerevisiae* connected to the reduction of the compound toxicity. The chromosome of *Escherichia coli* K-12 also bears the gene encoding OYE homologue NemA, an N-ethylmaleimide reductase, which blocks the formation of toxic products via modification of cysteine residues [15]. Further research of OYEs can therefore lead to the development of drugs avoiding pathogen resistance by interfering with their ability to biotransform xenobiotics [13].

OYEs share the same molecular architecture and have similar catalytic mechanisms but they play various biosynthetic roles in different species. Bacteria, fungi, protists, and plants usually code for more than one OYE but their transcriptional control differs so they cannot be classified as isoenzymes [16]. All known OYE’s enzymatic reactions suggest their broad substrate specificity [10]. The discovery of the morphine biodegradation pathway in *Pseudomonas putida* M10 [17] uncovered the participation of OYE morphinone reductase MorB in the second step of the degradation. Morphine catabolism has also been detected in *Agrobacterium* sp. R89-1 (CCM 7949), the strain we later formally described as *A*. *bohemicum* R89-1 [18]. We reported a hydroxylation of codeine at the carbon C14 [19], and a later genomic and biochemical study identified the OYE enzyme currently named XdpB. XdpB has the activity of morphinone reductase and catalyzes hydrogenation of the codeinone C7-C8 double bond [20]. Codeinone is an α, β-unsaturated ketone, that can act as a strong acceptor in Michael addition reactions causing the inactivation of biomolecules with thiol groups. Interestingly, this toxic intermediate plays a central role in the morphine/codeine biodegradatory pathway [21]. The discovery of the XdpB morphinone reductase activity and its ability to protect *A*. *bohemicum* R89-1 cells against codeinone toxicity spurred our further interest in XdpB and its role in morphine metabolism, degradation, and physiology.

Here we report the crystal structure of XdpB. Its closest homologues with the known structures are glycerol trinitrate reductase (GTN) NerA from *Agrobacterium radiobacter* [22] (UniProt O31246, 371 amino acid residues, identity of 56% and positivity of 71% with XdpB) and morphinone reductase MorB from *Pseudomonas putida* M10 (UniProt Q51990, 51% identity and 63% positivity, [17]). Despite the overall similarity of these three and other OYE structures, the XdpB structure differs by unexpected structural features with potential functional consequences. Some of these structural features led to the hypothesis of OYE enzymatic regulation which we tested by biochemical experiments and a bioinformatic analysis which are both reported in this work.

## Methods

### Media and culture conditions

The cultures of bacteria were grown in an LB medium (1.0% tryptone, 0.5% yeast extract, 1.0% sodium chloride; pH 7.0 adjusted before sterilization) or in an expression LB medium supplemented with trace elements—LBTE (TE: MgSO_4_.7H_2_O 200 mg.l^-1^, CaCl_2_.2H_2_O 50 mg.l^-1^ and FeSO_4_.7H_2_O 10 mg.l^-1^). The expression cultures were grown in 1 L Erlenmeyer flasks with 200 ml of the LBTE medium in a Max Q 4000 shaker, Barnstead, Lab-Line (250 rpm, 30°C). At OD600 of 0.4–0.6, the cultivation temperature was lowered to 16 °C (the host BL21(DE3)) or 10 °C (the host ArcticExpress (DE3)), the heterologous expression was induced by 1 mM IPTG and growth continued for 16 h (BL21(DE3)) or 24 h (ArcticExpress (DE3)). Strain BL21 (DE3) bearing a Takara Chaperone Plasmid Set (Takara) was used according to the manufacturer´s manual.

### Construction of the expression systems

The chromosomal DNA was isolated from the overnight culture of *A*. *bohemicum* R89-1 by the High Pure PCR Template Preparation Kit (Roche). The genes were cloned by the standard pET plasmids based workflow. All primers are listed in the Table A in S1 File. Genes were amplified by the PCR reaction with the primer pair, the specific product was separated by electrophoresis, double-cut by corresponding restriction enzymes, purified (QIAquick) and ligated by the T4 DNA ligase (NEB) into a pre-cleaved vector. The ligation mixture was used to transform deep-frozen competent cells of *E*. *coli* XL1 Blue and the culture was plated on an LB agar supplemented with kanamycin. This procedure led to new plasmids whose sequences were verified by sequencing. This procedure was used to produce plasmids bearing *XdpB*, *XdpA* and *XdpR* variants with His tag on the C (pET26b) or N (pET28b) terminal and tag-free proteins. The plasmid from a single colony isolate, bearing the required and verified fragment, was isolated by means of the QIAprep Miniprep Kit and used to transform expression hosts.

### Enzyme purification

The two-step purifications were based on IMAC and gel filtration chromatography. Cells were disintegrated by sonication (Sonicator 3000, Misonix, 15-20W) in a phosphate buffer (50 mM Na_2_HPO_4_, 150 mM NaCl, 0.2 mM TCEP, pH 8.0) with addition of 5 U/ml Benzonase Nuclease, a soluble fraction was separated by centrifugation (40 000 g, 4 °C, 30 min) and directly loaded on a gravity-flow NiNTA column (HisPur Ni-NTA Superflow Agarose). Pooled fractions with XdpB were finally purified in a HEPES buffer (20 mM HEPES, 20 mM NaCl, pH 8.0) with a HiLoad 16/600 Superdex 75 gel chromatography (NGC Chromatograph, Bio-Rad) to a single band purity on SDS-PAGE. The effect of His tagging on activities of the proteins was studied with crude enzyme solutions.

### Crystallization and structure determination

Crystals of diffraction quality were obtained by the hanging drop vapor diffusion method at 8 °C: the drops were composed of 2 μl of a protein solution and 2 μl of a non-buffered precipitant solution (50 mM tri-Lithium citrate, 32% (w/v) PEG 3350). Crystals were cryoprotected by 10% glycerol and flash frozen in liquid nitrogen. The diffraction data were collected at 100 K using the MX 14.2 beamline (BESSY II, Helmholz-Zentrum Berlin, Germany). The diffraction data set was processed using the XDS program package [23], scaling by Aimless from the CCP4 program package [24]. The structure was solved with the program BALBES [25]. Refinement was carried out with REFMAC5 [26] and manual corrections were performed using COOT [27]. The coordinates and structure factors have been deposited in the PDB with the accession code 5epd. The data collection statistics and refinement parameters are listed in Table 1 and the raw diffraction data have been deposited in the Zenodo repository under DOI: 10.5281/zenodo.250362.

**Table 1:** Crystallographic and refinement parameters of the XdpB structure as captured in the PDB entry 5epd.

Beamline	HZB, Bessy II, MX 14.2
Wavelength (Å)	0.91841
Resolution range (Å)	47.36–2.10 (2.16–2.10)
Space Group	P 21 21 21
Cell parameters (Å)	54.88, 68.60, 93.68
No. of observations	74,784 (4,185)
No. of unique reflections	20,170 (1,469)
Data completeness	94%
Multiplicity	3.7 (2.8)
Mean I/σ(I)	13.0 (2.1)
R_merge_	0.068 (0.358)
CC_1/2_	99.7 (82.8)
Wilson B value (Å^2^)	19.7
Refinement	
Reflections: working/free	19,114/1,012
Protein atoms	2632
Waters	221
R_work_/R_free_	0.198/0.262 (*)
R_all_	0.201
Ramachandran plot	342/348 (98%)
favored	330 (96%)
allowed	12 (4%)
disallowed	0
R. m. s. bond distance deviation (Å)	0.011
R. m. s. bond angle deviation (°)	1.417
Mean B factors: protein/solvent/overall (Å^2^)	37/38/37
PDB accession code	5epd

* Rfree from the PDB validation report is biased by using all reflections in our last run of structure refinement.

### Biochemical characterization of XdpB

Enzyme kinetics was studied with the *in vitro* flavinated protein to achieve its high enzymatic activity [22] and data were recalculated to XdpB saturated by FMN. The kinetic parameters of the enzyme were determined spectrophotometrically at the wavelength of 340 nm in the reaction mixture containing NADH (concentrations ranging from 0.015 to 0.3 mM), substrates (0.3 to 1 mM), a 50 mM phosphate buffer (pH 8) at a temperature of 25 °C. To calculate Km, the Lineweaver-Burk plot was used analogously to the protocol for morphinone reductase of *P*. *putida* M10 [18]. The optimum of the enzyme activity was determined in a 50 mM phosphate buffer with pH adjusted by citrate (the range from 4.8 to 9.0) and a 30 minute preincubation at a temperature ranging from 15 to 30 °C. The specific activity (SA) was assayed at a temperature of 25 °C with a 1mM substrate and 1 mM NADH. The activity is expressed in U.mg^-1^ protein. The activity of 1 U is the amount of enzyme oxidizing 1 μmole of NADH per minute. Protein concentration was assayed by the BCA Protein Assay Kit (Pierce) and verified by the Direct Detect infrared spectrometer (Merck). Bovine serum albumin was used as a standard.

### Biophysical characterization of XdpB

The oligomeric state was assayed on a Yarra SEC3000 3u column (Phenomenex, HPLC) and Superdex 75 16 600 columns. The aggregation of XdpB was determined by Dynamic Light Scattering (Zetasizer 90, Malvern) between 5 to 40°C with the protein solution (0.3 mg.ml^-1^ to 20 mg.ml^-1^). The circular dichroism (CD) spectra were measured by the Chirascan-plus spectrometer (Applied Photophysics) with wavelength steps of 1 nm (range of 185–260 nm) at 20 °C and a protein concentration of 0.2 mg.ml^-1^ [28]. The buffer subtracted spectrum was analyzed by the CDNN program. Temperature induced protein unfolding was measured by detecting the change of the ratio of tryptophan fluorescence at emission wavelengths of 330 and 350 nm between 20 °C and 95 °C (step 1 °C/min) using Prometheus NT.48 with UV capillaries (NanoTemper Technologies) loaded by 10 μl of protein sample per capillary; the FMN-free XdpB wild type had a concentration of 3.3 mg/ml, and the flavinated form 2 mg/ml.

### MicroScale Thermophoresis (MST)

The MST experiments were performed on a Monolith NT.115 instrument using 20% LED and 40% or 80% MST laser power, and hydrophilic capillaries. The proteins were labeled by the RED-NHS (Amine Reactive) kit according to manufacturer’s instructions and the excess of the dye was removed with the manufacturer-supplied dye removal columns. The labeling and measurements were done in a HNT buffer at 25 °C. The labeled XdpB-647 protein was measured in a 20 mM HEPES buffer at concentration of 200 nM. Data from three independently pipetted measurements for each experiment were analyzed by the MO.Affinity Analysis software version 2.1.2030.

#### Gel-shift assay of the XdpR transcription regulator

The regulatory segment of XdpR from the *A*. *bohemicum* R89-1 genome [19] was a short double-stranded DNA probe of 26 nucleotides obtained as complementary annealed primers (Fwd: CAATCATGATGATCGTCATGAATATA) with the negative control mutated (Fwd: CAATTATGATGATCGTTATGAATATA). The XdpR protein with an N terminal His tag was purified as described above for XdpB, concentrated to 0.5 mg/ml and preincubated with DNA (20 μM) for 1 h at room temperature. Electrophoretic separations were done in 7% PAGE gels in a blue native arrangement [29]. Precision plus protein All Blue Prestained protein standards (Bio Rad) and XdpR with 1% deoxycholate were used as markers. The gel was stained with ethidium bromide (1 μg/ml) in a TAE buffer for 2 h and destained for 30 min. Ethidium bromide fluorescence was recorded by MF-ChemiBIS 2.0 (DNR Bio-Imaging Systems).

### Reverse transcriptase qPCR

The primers were designed by means of the RealTimeDesign software (LGC Biosearch Technologies, USA) and all primers are listed in the Table B in S1 File. Primer functionalities and their concentrations (100 to 300 nM) were tested with the chromosomal DNA isolated from *A*. *bohemicum* R89-1 (for isolation protocol see Expression system construction) diluted to 2 ng DNA/μl in the PCR reaction: 95 °C 2 min for the initial denaturation and 40 times polymerization cycle of 95 °C 15 s, 60 °C 45 s, 72 °C 30 s. The polymerase master mix 2x PCRBIO HS Taq Mix Red (PCR Biosystems) was used. RNA was isolated by means of the High Pure RNA Isolation Kit (Roche) from the culture biomass of *A*. *bohemicum* R89-1 grown in 50 ml of an LB medium or the same culture biomass supplemented with codeine phosphate (final concentration of 1g/L) from exponential, early and late stationary growth phases. cDNA was synthesized by the Transcriptor First Strand cDNA Synthesis Kit (Roche) on all isolated RNA templates. Concentration of cDNA was determined with Nanodrop 100 (Thermo Scientific) and diluted to the final concentration of 2 ng cDNA/μl. The qPCR reaction was performed using the StepOnePlus Real-Time PCR System (Applied Biosystems) with the SYBR Select Master Mix (Applied Biosystems) in the reaction mixture (volume of 20 μl) containing 4 ng cDNA and primers at a concentration of 200 nM. The reaction cycle: 2 min 50 °C, 2 min 95 °C, followed by 40 times (95 °C 15 s and 60 °C 1 min). Data analysis was performed by the StepOne Software (Applied Biosystems), using the the Relative Quantitation/Comparative CT (ΔΔCT) setting. The sample of culture LB0 grown in an LB medium (13.5 h) was used as a reference and the *coxA* gene served as an endogenous control. All experiments were done in independent triplicates.

### Bioinformatic analysis

The UniProt and PDB databases were searched with the amino acid sequence of XdpB as a query to select 150 sequences that were subjected to a multiple sequence alignment (MSA) by the ClustalW algorithm as implemented in the Ugene program [30] or MEGA 6 [31]. Their genomic localization in the *xdo* operon was verified by the tblastn tool (Blast, NCBI, version (2.6.0)). Structural comparison was prepared by MatchMaker implemented in the UCSF Chimera software (version 1.10.2, Needleman-Wunsch algorithm, BLOSUM-62 matrix) [32]. The binding of the C-terminal pentapeptide TTSDN in the active site of the symmetry-related molecule was analyzed using LigPlot+ [33] with the symmetry-related pentapeptide as a ligand. GenBank was searched by the blastp and DELTA blast tool [34]. Bacterial Operon and Gene Prediction was done by the Software FGENESB [35]. Operator-like sequence characteristics for the TetR family proteins were determined based on the results obtained by Deng at al. [36] and promoters were analyzed by the MacLellan's method [37].

### The docking computations

The FMN molecule and respective peptides were docked into the crystal structures of PDB codes 2hsa and 5epd using AutoDock Vina [38]. The docking parameters were prepared using the Python Molecular Viewer (PMV 1.5.6 rc3; [39]). Each ligand was allowed to sample docking poses in a 40 Å x 40 Å x 40 Å box centered around the entrance to the ligand binding cavity.

## Results and discussion

### Overall structure of XdpB

**Fig 1:**
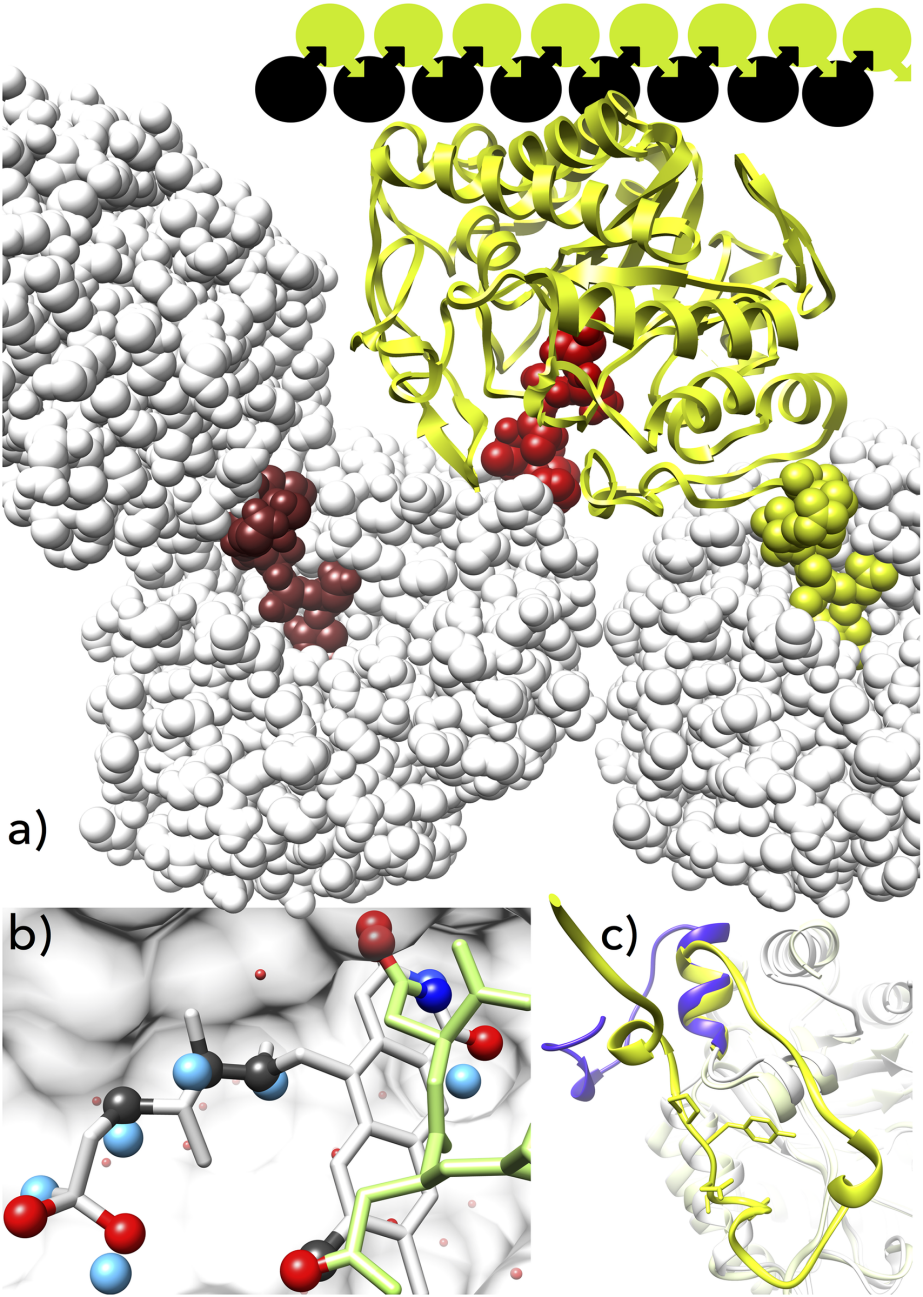
Structure of the XdpB, an old yellow enzyme.

We solved the crystal structure of a new monomeric OYE enzyme XdpB from *A*. *bohemicum* R89-1 (Fig 1) at 2.1 Å crystallographic resolution in the space group P2_1_2_1_2_1_ with one protein chain in the asymmetric unit. Data collection statistics and other crystallographic data are summarized in Table 1 together with a summary of structure validation. The electron density map was interpreted starting at the fifth residue for the whole protein except for two loops (G267—A276, and N295—F297). The core of the structure is formed by the TIM barrel fold with eight parallel β-strands surrounded by eight α-helices (Fig 1a). The flavin mononucleotide (FMN) was not observed in the electron density.

XdpB is a close homologue of NerA (PDB ID 4jic [22]) with the root-mean-square-deviation (rmsd) of 0.72 Å calculated between 321 CA atom pairs and 1.65 Å across all 342 pairs; their FMN binding site residues superimpose even closer (the zone closer than 5 Å from FMN with 20 atom pairs has rmsd 0.28 Å). The active site pocket with Y181, Y68, F345, and catalytic residues H176 and N179 is arranged similarly in both structures. A comparison of flexibilities of the XdpB and NerA crystal structures by juxtaposing their scaled B-factors [40] showed that the former is more flexible in agreement with its more low-temperature tolerant character. Two parts of XdpB, the β-hairpin lid of the TIM barrel and the N-terminal cap are, however, less flexible than in NerA (Figure A in S1 File). Another close homologue of XdpB, morphinone reductase MorB (PDB 1gwj, [41]) is the first enzyme known to reduce morphinone. Its structural similarity is comparable to that of NerA; rmsd calculated for 319 atom pairs is 0.78 Å (across all 346 pairs: 2.37 Å). The active sites in XdpB and MorB are highly similar with the exception of Y181 in XdpB being replaced by C191 in MorB.

### Interactions of XdpB C-terminal amino acids with the FMN binding site

The XdpB structure revealed that the putative FMN binding site of one XdpB molecule is occupied by the C-terminus of a symmetry-related XdpB molecule (labeled by *) arranging XdpB monomers into infinite linear chains (Fig 1a). The putative FMN position in XdpB was deduced by aligning XdpB, NerA, and MorB structures (Figure B in S1 File). In XdpB, FMN is replaced by side chain atoms of the symmetry-related Asp364* and Asn365*: the position of the C-terminal residue of the symmetry-related XdpB, Asn365*, is fixed by hydrogen bonds to the active site residues Tyr181, His176, Gln100, and Arg228; the interactions are depicted in the Figure C in S1 File as a Ligplot+ schema. The Asn365* side chain atoms are stabilized by interactions with Gln100 and replace hydrogen-bonding FMN atoms N3, C2 and O2. Asp364* is stabilized in its position by hydrogen bonding with Arg318 and partially fills in the position of the aromatic flavin ring. The rest of the putative FMN binding site, which would be occupied by ribitol and phosphate, is occupied by five water molecules.

The amino acids lining the putative FMN site form a complex network of interactions with the C-terminal region of the symmetry-related XdpB with the surface of 530 Å^2^: 10 hydrogen bonds, 4 water bridges, one CH-π interaction (interactions between the FMN binding site and the C terminus are highlighted in the Figure C in S1 File). The observed intermolecular interaction between two XdpB molecules prevents the binding of FMN and we therefore propose that the interaction may represent a mechanism of self-inhibitory regulation of the XdpB activity (Fig 1b). We hypothesize that the catenation of XdpB molecules observed in the crystal structure acts as a concentration dependent self-regulation. Self-inhibition and modulation of protein activity by intramolecular and intermolecular blocking mechanisms is common in proteases, kinases, and receptors [42, 43], but rare in OYE flavoproteins [44]. The self-inhibitory blocking by defined protein oligomerization is quite unique and to the best of our knowledge has not been reported yet. The self-inhibitory blocking of OYEs is further discussed below in context of their sequence analysis and genomic organization.

The interaction potentially leading to self-inhibitory blocking has also been observed in the crystal structure of OPR3, OYE from tomato *Solanum lycopersicum* (39% of sequence similarity to XdpB, PDB code 2hsa [44]). The mechanism of blocking in OPR3 is however completely different from that in XdpB: two OPR3 molecules mutually block their active (not FMN) sites by binding their neighbor’s loop Q289-A292 (Loop L6) forming thus the homodimer. The authors of the OPR3 structure reported the equilibrium dissociation constant of dimerization Kd = 30 μM. The protruding loop L6 is also present in another OPR3 structure from *Arabidopsis thaliana*, but dimerization is not observed in this crystal structure (PDB code 1q45 [45]). Contrary to OPR3, the XdpB self-interaction is asymmetric and cannot lead to complete inactivation of the protein.

### The extent of XdpB flavination and its oligomerization

The extent of XdpB oligomerization depended on the expression system and construct. A construct with the N-terminal his tag (designated as XdpBwt) overexpressed in *E*. *coli* oligomerized and the amount of FMN in the purified enzyme sample did not exceed 20% of the molar equivalent. On the other side, variants with the C-terminal his tag (XdpB Chis) or the last 5 amino acids deleted (XdpBΔ5) did not oligomerize, and at the same time, they showed the highest molar percentage of XdpB flavination (up to 80%, details in Text A in S1 File). Direct addition of 5 mM riboflavin, an FMN precursor [46], to the XdpBwt producing culture increased the flavin content in XdpBwt by only 10 to 15% showing that the FMN synthesis is not the rate limiting step critical for the FMN content in the expressed XdpB. The only way to increase saturation of XdpBwt by FMN above 80% was by mixing purified protein with a 10 mM FMN solution at 4 °C overnight and removing the excess of FMN by size exclusion chromatography.

Observations from the chromatography and DLS experiments demonstrate the formation of oligomers of XdpBwt in a solution. No similar effect was observed for XdpBΔ5 and XdpB Chis. The difference of the elution volume between the XdpBwt and XdpBΔ5 variant on gel filtration chromatography (Superdex 75 16/600) was large, nearly 4 ml, which corresponded to a mass shift of 10 kDa (the expected shift was negligible: -536 g/mol for TTSDN peptide and +456 g/mol for FMN, Figure D in S1 File). The molecular diameter of XdpBwt measured as dynamic light scattering z-average is concentration dependent and shows a much larger diameter (23 ±5 nm) than the expected dimensions of the monomeric XdpB in the crystal structure (8–12 nm). The DLS measured dimensions of XdpBwt are concentration dependent and the extent of oligomerization is hard to estimate. We assume that it occurs via the concatenation mechanism observed in the crystal structure. The DLS diameter for XdpBΔ5 (15 ±2 nm) is in the rank of the monomer rather than the dimeric or higher order assembly, and is not concentration dependent. The aggregation of the XdpBwt started at a temperature of 28 °C as determined by DLS and the aggregation seems to be partially reversible by sample cooling. All the phenomena described above were demonstrably suppressed by titration of the enzyme solution by FMN.

**Fig 2:**
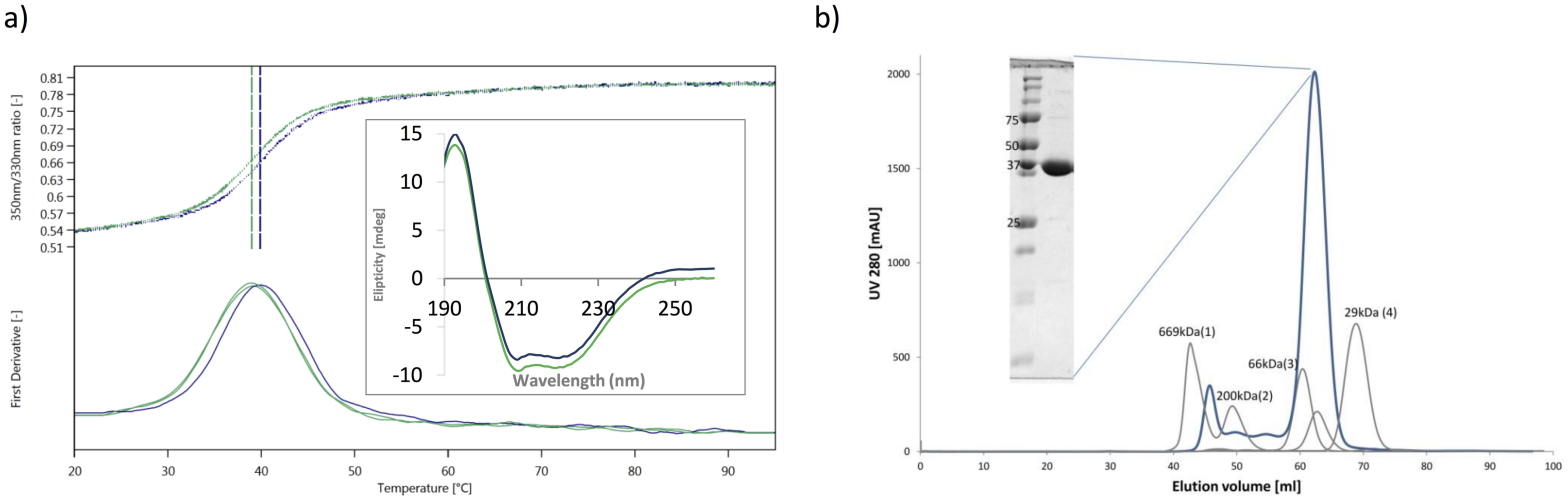
The biophysical properties of XdpB.

To exclude a possibility that the observed effects are due to poor protein quality, we analyzed the melting curves measured by CD spectra of both apo and FMN-bound protein forms. The melting temperatures of 38.9 and 39.8 °C were determined for the XdpBwt apo form and *in vitro* flavinated form, respectively by nanoDSF. Also, CD spectra of both enzyme forms are virtually identical suggesting their identical fold (Fig 2a). Neither method thus indicated protein unfolding and the observed aggregation of the XdpBwt apo form cannot be attributed to protein denaturation. We do not know whether the FMN-bound or FMN-free (inactive) XdpB exists in the native host, *A*. *bohemicum* R89-1, because we do not know the concentrations of XdpB and FMN inside the bacteria.

Possible binding between the C-terminal TTSDN pentapeptide and the FMN site was tested by MST experiments. We measured the affinity between a synthetic TTSDN peptide (98% purity, Thermo, maximal concentration 2.5 mM) and a XdpBΔ5 protein, which had the C-terminal TTSDN deleted. Depending on the exact composition of the protein solution and XdpB preparation history, the equilibrium constant of TTSDN dissociation from XdpBΔ5 was evaluated between 10^−5^ and 10^−7^ M; an example measurement is shown in Figure C in S1 File. The reported dissociation constants for forming the OPR2 homodimer (3x10^-5^ M [44]) and binding between the FMN and an apo form of NerA (10^−7^ M [22]) frame the range of our measurements. XdpBΔ5 oversaturated by 1mM FMN showed no detectable affinity to the TTSDN peptide. This negative control excluded the possibility that the observed interaction resulted from a nonspecific peptide binding to XdpB and supported the validity of the crystallographically observed binding between the symmetry-related C-terminal TTSDN and FMN binding site.

The presented experimental data are supported by results of our docking computations performed on the crystal structures of OPR3, PDB code 2hsa, and of XdpB, code 5epd. The interactions in 5epd predicted by the docking software and for 2hsa are shown in Figure E in S1 File as plotted by LigPlot+. Docking mimicking the blocking of XdpB by peptide TTSDN and of OPR3 by peptide VAYGQTEAGRLGS showed the same binding energy of about -5.3 kcal/mol. The FMN binds to both proteins more strongly, ΔG values are -8.8 kcal/mol for XdpB, and -10.9 kcal/mol for 2hsa. Because binding energies of the C-terminal peptides and FMN are comparable, the blocking in these two enzymes is most likely competitive and dependent on local concentrations of the enzymes and FMN.

### XdpB kinetics

We classified XdpB as a glycerol trinitrate reductase with maximum activity at a temperature of 25 °C and pH between 8 and 9. As determined by the ThermoFluor method XdpB is most stable in a HEPES buffer pH 8 (100 mM) and NaCl concentration 200 mM. Under these conditions, the k_cat_ of XdpB for GTN equals 7.4 ±1.2 s^-1^, K_m_^GTN^ = 460 ±132 μM, and K_m_^GTN^/K_m_^NADH^ = 55–85. The K_m_^GTN^ value corresponds to that of NerA (705 ±74 μM [47]). The K_m_^NADH^ equals 6 ±1 μM. The specific activities (pH 8, 25 °C) for other substrates are as follows: codeinone 15.3 ± 0.7 U.mg^-1^, morphinone 6.5 ±0.4 U.mg^-1^, and cyclohexenone 4.5 ±1 U.mg^-1^. These measurements showed that XdpB and NerA [22] have similar kinetic parameters. Because amino acids involved in the catalysis and cofactor binding are highly conserved between the two enzymes, we concluded that both belonged to the same kinetic group [48].

### The classification of OYEs according to their C-terminus

Unusual structural features of XdpB prompted us to investigate sequences, structures, and genomics of OYEs in order to find whether the phenomenon of self-inhibitory blocking is to be expected in other OYEs as well. The structure-based sequence alignment of 17 available OYEs structures from different organisms (Table 2) showed that they were highly similar except for their C-termini, which showed significant differences. Based on these differences, we were able to discriminate two OYE subgroups, called hereafter C1 and C2. The C1 subgroup is epitomized by XdpB and characteristic by a strictly conserved GxxDYP motif just preceding the C terminus. We hypothesize that this motif determines the proper orientation of the C terminus (Fig 1c) and is thus a prerequisite for self-inhibition (other features giving rise to self-inhibition are discussed below).

**Table 2:** Selected OYE enzymes.

OYE C1 family: protein, organism	PDB	Activity GTN [U.mg^-1^]	°C	Reference
XdpB, *Agrobacterium bohemicum* R89-1	5epd	2.5 ± 0.3**	18	This work
OYE1, *Saccharomyces pastorianus*	1bwk	-	-	[59]
AtOPR3, *Arabidopsis thaliana*	1q45	-	-	[45]
PETN, *Enterobacter cloacae* PB2	1h51	12.1 ± 0.5	Rt	[60,61]
SYE, *Shewanella oneidensis*	2gou	-	-	[55]
OPR3, *Solanum lycopersicum*	2hsa	-	-	[44]
TcOYE, *Trypanosoma cruzi*	3aty	-	-	[5]
Ncr, *Zymomonas mobilis*	4a3u	-	-	[62]
NerA, *Agrobacterium radiobacter*	4jic	3.1 ± 0.1	30	[22]
EasA, *Aspergilus fumigatus*	4qnw	-	-	[56]
MorB, *Pseudomonas putida*	1gwj	-	-	[41]
GTN, *Agrobacterium radiobacter*	-	15.0*	Rt	[64]
OYE C2 family: Protein, organism	PDB	Activity GTN	°C	Reference
YqjM, *Bacillus subtilis*	1z41	-	-	[48]
TOYE, *Thermoana*. *pseudoethanol*. E39	3kru	-	-	[49]
XenA, *Pseudomonas putida* 86	3l5l	-	-	[63,]^65^, ^66^]
*Pseudomonas putida* II-B, *P*. *fluorescens* I-C		124 ± 6	25	
OYE, *Geobacillus kaustophilus*	3gr8	-	-	[67]

* The activity was assayed with GTN in absence of NA(P)DH.

** The activity recalculated to XdpB fully saturated with FMN.

The C2 subgroup is characterized by a higher sequence and structural plasticity compared to the C1 subgroup but their C termini contain a structurally defined motif with a consensus sequence PxxY. It adopts structures that are directly responsible for the dimerization or tetramerization necessary for the formation of active sites [8,49,50] (Fig 1c). Members of the C2 subgroup occur predominantly in G+ bacteria, are well characterized regarding their transcription and translation and are, in certain cases, related to oxidative stress: e.g., the YqjM from *Bacillus subtilis* [8], SYE4 from *Shewanella oneidensis* [16], and CyeR from *Corynebacterium glutamicum* [51]. As is evident from the subgroups´ crystal structures, the C terminal sequences of both subgroups are involved in interactions between OYE subunits and in the formation of active sites.

Sequence alignment identified the 150 OYE enzymes homologous to XdpB and therefore belonging to the C1 subgroup. A majority of them were annotated as glycerol trinitrate reductases, N-maleimide reductases, or alkene reductases. Literature data suggest a possible role of some C1 members in apoptosis [9], alcohol stress [52], or the oxidative stress response [53,54], but XdpB (see below) and SYE2 of *Shewanella oneidensis* [55] are not upregulated at the oxidative stress response. This indicates the involvement of OYE C1 members in multiple pathways rather than a physiological activity in one process only.

In bacterial species, the GxxDYP motif (Table C and Figure F in S1 File) of the C1 OYEs is followed by up to twelve amino acids, predominantly G, A, L, S, T, N, and D. The forming of homodimers in OPR3 [44] and linear chains in XdpB indicates at least two autoinhibitory mechanisms, both of which are achieved by the insertion of loops bordering the active site (loop L6 in [44], Figure F in S1 File). However, self-inhibition was not observed in the other bacterial OYE with known structures. One explanation may be that a majority of them had been heterologously produced with C-terminal his tags so that the interaction leading to self-inhibition was disabled. This is the case of e.g. NerA, EasA from *Aspergillus fumigatus* (PDB ID 4qnw [56]), GYE from *Gluconobacter oxydans* (PDB ID 3wjs).

### The bioinformatic analysis of the xdo operon

**Fig 3:**
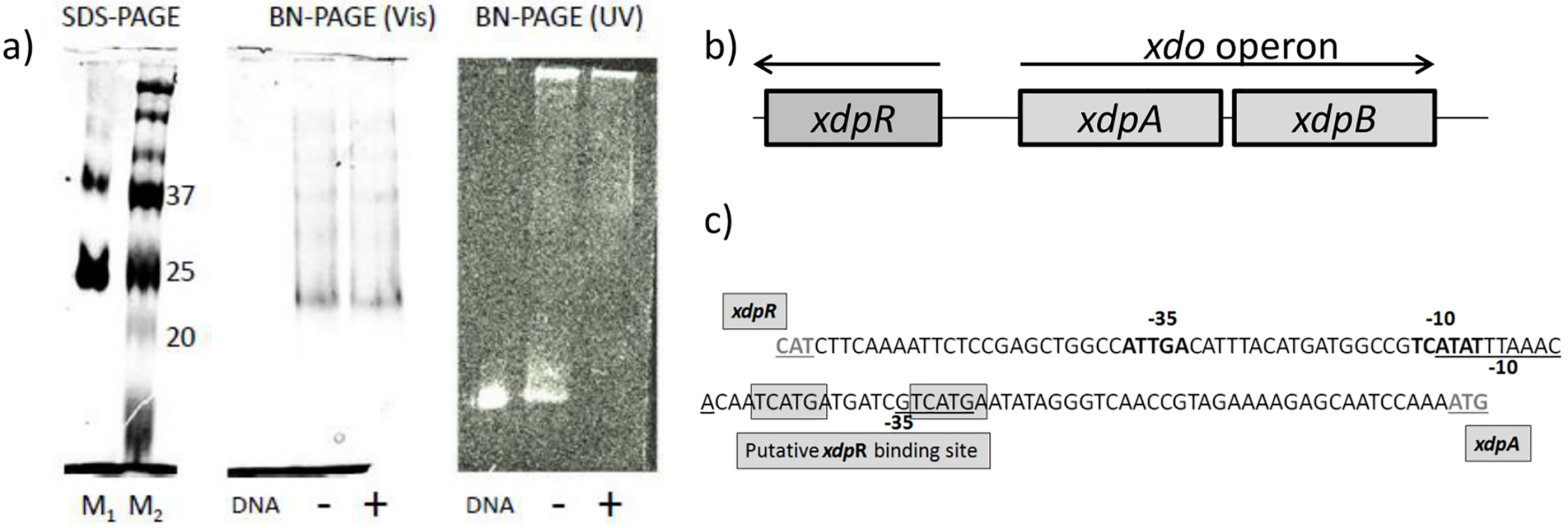
The gel-shift assay of the *xdo* operon.

To identify genomically relevant features common to the C1 OYEs, we investigated the *xdpB* gene organization and regulation of the strain R89-1. It revealed that *xdpB* forms an operon with the gene *xdpA* coding short chain dehydrogenase; the operon was denoted as *xdo*. A DNA segment bearing the *xdo* operon was obtained together with flanking sequences by PCR techniques, sequenced (access number KM272590), and its nucleotide sequence was verified by the Ilumina whole genome sequencing (access number LNUW00000000 [20]). We found that the operon was controlled by a transcriptional regulator from the TetR family that precedes *xdo* in the reverse orientation; the regulator was named *xdpR* (Fig 3b). The *xdo* operon is widespread mainly among *Proteobacteria*: its single copy is present in genomes of several bacteria, e. g. *Rahnella*, *Enterobacter*, *Pseudomonas*, *Serratia*, *Xanthomonas*, and *Stenotrophomonas*. The operon was also identified on plasmid pTiBo542 of *Agrobacterium tumefaciens* (GenBank: DQ058764). In all of these cases, the *xdo* operon consists of tandem genes encoding OYE plus a short chain dehydrogenase, aldehyde dehydrogenase (*Polaromonas naphthalnivorans* CJ2; NC_008781), or Zn-alcohol dehydrogenase (*Ralstonia pickettii* 12J; CP001068) and is regulated by the TetR family transcriptional regulator. Due to genomic organization, we expect that the enzymes coded by *xdo* take part in a single metabolic pathway. We hypothesize that the *xdo* coded dehydrogenase mediates the reduction of alcohol into α, β-unsaturated aldehyde or ketone that are substrates for OYE.

OYE C1 proteins are coded in the *xdo*-like operon or are under control of different types of transcription regulation. Only the enzymes located at *xdo* are likely to express the self-inhibitory blocking of their FMN binding sites because they are terminated by polar amino acid residues, which can compete with FMN for the FMN binding pocket. Enzymes under different control than the TetR family regulator end predominantly with hydrophobic residues, which cannot bind to the FMN site (Table C in S1 File). To support this hypothesis, we performed docking calculations with two OYE C1 members with genes located in *xdo* (UniProt ID: A0A077LI68 and D8G840) and one OYE C1, which is not located in *xdo* (UniProt ID: A0A0D2TBF3). The results of these calculations confirmed the view that only the C terminus of the former proteins is prone to specific interaction with the FMN binding site (Figure G in S1 File). Different regulation mechanisms of genes of the OYE C1 subgroup represent a new evidence for the hypothesis that these enzymes play roles in multiple metabolic pathways.

### Biochemical characterization of XdpA and XdpR

Because XdpA, XdpB, and XdpR proteins are members of one transcription unit, their detailed analysis may help to understand the physiological role of XdpB. *XdpA* and *xdpR* genes were independently cloned into expression vectors and recombinant plasmids were obtained, expressed in the *E*. *coli* ArcticExpress (DE3) host and characterized as described below.

### XdpA is short chain dehydrogenase

A BLAST search was not unequivocal but after a manual sequence revision of 50 orthologues of short chain dehydrogenase (SDR) and 50 orthologues of the AraC family, we found out that the XdpA protein belonged to the NADB Rossman superfamily and was SDR [57]. A simplified form of the alignment is shown in the Figure H in S1 File. SDS PAGE (Figure I in S1 File) and gel filtration chromatography showed that XdpA was a homodimer composed of two subunits of 262 residues, molecular weight of 27.6 kDa, and melting temperature of 38.5 °C. The purified protein quickly aggregated at room temperature and psychrophilic behavior is expected. We failed to identify enzymatic activity of XdpA to substrates associated with the metabolism of codeine, namely codeine, morphine, croton aldehyde, allyl alcohol, n-butanol, isobutanol, cyclohexanol, or 4-hydroxybenzoic acid in a phosphate buffer system (pH 6–8) supplemented with NAD^+^ or NADP^+^. It is therefore likely that the substrate spectrum for XdpA is more restrictive than that for OYE proteins.

### The XdpR protein regulates the transcription of the xdo operon

We predicted the transcriptional regulation of the *xdo* operon by the XdpR regulator using a bioinformatic analysis and verified the prediction by an *in vitro* gel-shift assay. The promotor-transcriptional start sequences of the *xdo* operon and *xdpR* gene were analyzed by the MacLellan method [37] together with an identification of an operator-like DNA sequence recognizable by the TetR family of proteins [36]. The results are schematically represented in Fig 3c: the nucleotide sequence recognized by the transcription regulator XdpR was predicted to be TCATGAXXXXXXTCATGA. This regulation sequence extends over the promoter regions of both *xdp*A and *xdp*R (Fig 3b) so that the expression of both proteins is negatively controlled by XdpR. The identical regulation sequence can be found in the genome of related *Agrobacteria sp*. C58 (gb|AE007872.2) and H13-3 (gb|CP002249.1) strains.

The XdpR regulator was expressed and purified (Figure I in S1 File). The gel filtration, as well as native and SDS electrophoretic experiments showed the XdpR preference for a dimeric state with a molecular mass of 37 kDa (Fig 3a). After the NiNTA chromatography, we observed rapid aggregation. The binding of XdpR to the operator site was confirmed by a gel-shift assay (Fig 3a). A negative control experiment, in which the DNA binding sequence was mutated, showed lower binding between the mutated DNA and XdpR protein. The role of XdpR as an effector molecule regulating *xdpA* and *xdpB* expression could therefore be expected.

### XdpB is not upregulated in oxidative stress

Because XdpB is OYE, we studied its involvement in the response to oxidative stress by using RT-qPCR of the relative gene transcription. The transcriptome of *A*. *bohemicum* R89-1 grown in an LB medium (a reference sample) was compared to that grown in an LB medium supplemented by codeine, a XdpB substrate, and a precursor of codeinone. Codeinone is a highly reactive α, β-unsaturated keton, a compound disrupting the cell redox state by Michael addition [58], and inducing oxidative stress. Our previous data indicated that codeinone, 14-OH-codeinone, and products of XdpB mediated conversion hydrocodone and oxycodone were present in *A*. *bohemicum* R89-1 cells after growth in an LB medium supplemented by codeine [19]. The RT-qPCR experiments with the *coxA* gene (Cytochrome c oxidase subunit) as an endogenous control detected similar concentrations of mRNA for *xdpR* and *xdpB* for both reference and sample implying similar transcription levels of XdpA and XdpB (Figure J in S1 File). At the same time, the same RT-qPCR experiments detected a significant upregulation of oxidative stress markers *kat*G coding peroxidase (concentration increased 9x), *trx*1 (coding thyoredoxin, 4x), and *gsh*B (glutathione synthetase, 4x). Our qPCR results thus indicate that the *xdo* operon is not involved in the physiological response to oxidative stress because neither this substrate nor oxidative stress induce *xdo* expression.

## Conclusions

We characterized the Old Yellow Enzyme XdpB from *Agrobacterium* sp. R89-1 as a glycerol trinitrate reductase by determining its sequence homology, substrate specificity, and 3D crystal structure. The XdpB structure was solved at 2.1 Å crystallographic resolution and deposited to the PDB under the code 5epd. Similarly to the other OYE proteins, the XdpB structure acquires the TIM barrel fold but it exhibits a unique structural feature when its FMN binding site is occupied by C-terminal residues of the TTSDN sequence from a symmetry-related molecule forming thus linear chains. The structure is the first published apo (FMN-free) form of OYE. Our biophysical experiments showed that XdpB can exist in both inactive FMN-free and FMN-bound forms depending on the XdpB and FMN concentrations, and that both of these forms have a similar stability.

Sequence and structure-based bioinformatics allowed us to discriminate two classes of OYEs, subgroups C1 and C2. In both subgroups, C-termini play important regulatory roles. Enzymes of the C1 subgroup, which is represented by XdpB, are active as monomers and can be identified by a conserved GxxDYP motif in the C-terminus proximity. The motif is a prerequisite of XdpB self-inhibition by binding of the TTSDN peptide to the FMN binding site of a neighboring molecule. An analogical self-inhibition by reversible and concentration-dependent oligomerization is expected to occur in the other C1 subgroup OYEs. The physiological function of the C1 subgroup enzymes is not clear, but as our results indicate, they are involved in several independent physiological pathways. In contrast to another OYE C1 member, N-ethylmaleimide reductase, our data indicate that XdpB was not involved in the response to oxidative stress.

Enzymes from the OYE C2 subgroup are characteristic by the C-terminal PxxY sequence motif. They are enzymatically active as homodimers or homotetramers. Di- or tetramerization is necessary to form FMN or active sites and is mediated by the specific interaction between the C-termini. These enzymes are involved in the cell response to oxidative stress, mostly of G+ bacteria.

Gene coding of XdpB is accompanied by a gene of oxidoreductase forming the *xdo* operon. This operon is conserved in a single copy in *Proteobacteria* and other bacterial species. The gel shift assay proved that the *xdo* operons are regulated by tetR-like repressors (designated as XdpR). We proposed that the whole operon is involved in a biochemical pathway dealing with a so far unidentified substrate. The pathway includes alcohol dehydrogenation by a short-chain dehydrogenase and a subsequent reduction of the product, unsaturated aldehyde or ketone, by OYE.

## Supporting information

S1 File All supplementary information (text A, tables A—C, and figures A—J) are included in this file. (DOCX)
