# Supplementary material for: The crystal structure of XdpB, the bacterial old yellow enzyme, in an FMN-free form
Source: PLoS One. 2018 Apr 9;13(4):e0195299. doi: 10.1371/journal.pone.0195299 (PMC5891007; doi:10.1371/journal.pone.0195299)
Supplement: S1 File — (DOCX) [file pone.0195299.s001.docx]

SUPPLEMENTARY MATERIAL

**The crystal structure of XdpB, the bacterial old yellow Enzyme, in an FMN-free form**

Jiří Zahradník ^(1)(2)(3) *^, Petr Kolenko ^(1)(4)^, Andrea Palyzová ^(3)^, Jiří Černý ^(1)^,

Lucie Kolářová ^(1)^, Eva Kyslíková ^(3)^, Helena Marešová ^(3)^, Michal Grulich ^(3)^,

Jaroslav Nunvar ^(1)^, Miroslav Šulc ^(2)^, Pavel Kyslík ^(3)^ and Bohdan Schneider ^(1) *^

(1) Institute of Biotechnology CAS, v. v. i., BIOCEV, Vestec, Prague West, Czech Republic

(2) Department of Biochemistry, Faculty of Science, Charles University Prague, Czech Republic

(3) Institute of Microbiology CAS, v. v. i., Prague, Czech Republic

(4) Dept. of Solid State Engineering, FNSPE CTU, Prague, Czech Republic

(*) The corresponding authors

E-mail: bohdan.schneider@gmail.com; zahradj@ibt.cas.cz

Running title: Bacterial Old Yellow Enzyme in an FMN-free form

Keywords: Old yellow enzyme; XdpB; X-ray structure; *Agrobacterium bohemicum* R89-1; self-inhibition; glycerol trinitrate reductase

**Abstract**

Old Yellow Enzymes (OYEs) are NAD(P)H dehydrogenases of not fully resolved physiological roles that are widespread among bacteria, plants, and fungi and have a great potential for biotechnological applications. We determined the apo form crystal structure of a member of the OYE class, glycerol trinitrate reductase XdpB, from *Agrobacterium* *bohemicum* R89-1 at 2.1 Å resolution. In agreement with the structures of the related bacterial OYEs, the structure revealed a TIM barrel fold with an N-terminal β-hairpin lid, but surprisingly, the structure did not contain its cofactor FMN. Its putative binding site was occupied by a pentapeptide TTSDN from the C-terminus of a symmetry related molecule. Biochemical experiments confirmed a specific concentration-dependent oligomerization and a low FMN content. The blocking of the FMN binding site can exist *in vivo* and regulates enzyme activity. Our bioinformatic analysis indicated that a similar self-inhibition could be expected in more OYEs which we designated as subgroup OYE C1. This subgroup is widespread among G-bacteria and can be recognized by the conserved sequence GxxDYP in proximity of the C termini. In proteobacteria, the C1 subgroup OYEs are typically coded in one operon with short-chain dehydrogenase. This operon is controlled by the tetR-like transcriptional regulator. OYEs coded in these operons are unlikely to be involved in the oxidative stress response as the other known members of the OYE family because no upregulation of XdpB was observed after exposing *A.* *bohemicum* R89-1 to oxidative stress.

**Text A. Supplementary methods and results**

**Enzyme heterologous expression and purification.** The FMN content in heterologously expressed XdpB depends on the construct used and the expression system. Variants with restricted C terminal interaction (missing or blocked (His tag) C terminus) always showed higher molar percentage of FMN in comparison with WT (N his). XdpBΔ5 and XdpBChis variant after purification from Escherichia coli BL21(DE3) (24 h, 16°C, induction 1 mM IPTG) contain 74 ±7 and 68 ±10 % FMN and XdpB Nhis produced under the same conditions showed only 32 ±10 % FMN. A high FMN content (60 ±10 %) was determined with XdpB Nhis culture of the host BL21(DE3) after induction with 1mM IPTG (20°C), but protein was mainly found in inclusion bodies under these conditions. The XdpB Nhis protein was completely soluble when synthesized at 10 °C in the strain Arctic Express(DE3), but the FMN content was only 8-17 % of the equimolar amount. XdpBΔ5 produced in the strain Arctic Express(DE3) at 10 °C contained 38 ±8 % FMN. Construct XdpB∆5 and XdpB∆5 CHis shows almost no difference in flavination. Two times increase of FMN content in all construct used was achieved by coexpression with groES/groEL chaperones (Takara chaperone plasmid set). This is together with direct supplementation of LB broth by FMN indirect proof that the FMN synthesis is not limiting for enzyme flavination. CD and melting for all purified XdpB variants were identical (Fig 2).

**Thermofluor assay.** The method was used to optimize a buffer pH and ionic strength by means of Tm determination using CFX384 Touch™ Real-Time PCR Detection System, Bio-Rad [S1]. The XdpB solution (1.2 mg.ml^-1^) was applied to Flat Cap strips (Bio-Rad) as follows: 6.25 µl and 3.13 µl of the solution was mixed with 17.75 µl and 20.87 µl of buffer, respectively. The SYPRO® Orange dye (Sigma) was used as fluorescent label (1 µl of 25 times diluted stock solution into each reaction mixture). The mixtures were pre-incubated for 30 min at 0 °C and the reaction temperature was step-wise increased by 0.5 °C in the range from 4 to 80 °C. Fluorescence of mixtures was assayed after 30 s incubation at each temperature in FRET mode and the results were evaluated (Bio-Rad CFX Manager 3.0). Parallel assays were performed using a method ThermoFAD (FMN) with samples prepared analogously [S2].

**Computer docking of selected OYE C1 proteins.** Protein selection: structures of the subgroup C1 encoded in *xdo* operon suitable for docking computations were not available on PDB. Therefore, we randomly selected two OYE C1 protein sequences, one from the group of proteins encoded in *xdo* operon and one with different transcription control. The selection was based on three parameters: The length of the C terminal tail of OYE C1 protein (after GxxDYP) at least eight residues long; the known genome of a source organism or close relative (allowing gene localization); maximum homology to XdpB 60 %. We selected Flavin oxidoreductase D8G840 (UniProt) from *Oscillatoria* sp. PCC 6506 encoded in *xdo* operon and 12-oxophytodienoate reductase A0A0D2TBF3 from *Skermanella aerolata* KACC 11604 (S7 Fig). Homologous model generation: The crystal structure of the XdpB (PDB ID 5epd) in interaction with symmetry related molecule was used as a template for homology modeling performed using the MODELLER 9v14 suite of programs [S3] with multiple sequence alignment in MUSCLE [S4]. The modeled proteins without the C terminal peptide have been overlaid over two XdpB molecules linked by the C-terminus of one of them. C terminal peptide docking computations: The missing C terminal peptide was generated by “loopmodel” function of the MODELLER with the series of 2 000 models generated for each structure. The 20 lowest energy models (sorted according to the DOPE score) were used for analysis.

**Table A. Expression system: construction of primers.**

| **Expression system construction primers** | | |
| --- | --- | --- |
| **Construct** | **Plasmid** | **Primers** |
| XdpB NHis | pET28b | fwd: TTTCATATGACCAAGACCACACTCTTCC,  rev: TTTGAATTCTCAATTGTCGGACGTTGTTT |
| XdpB CHis | pET26b | fwd: TTTCATATGACCAAGACCACACTCTTCC,  rev: TTTGAATTCATTGTCGGACGTTGTTT |
| XdpB WT | pET26b | fwd: TTTCATATGACCAAGACCACACTCTTCC,  rev: TTTGAATTCTCAATTGTCGGACGTTGTTT |
| XdpB∆5 | pET28b | Fwd: GATTTCATATGACCAAGACCACACTCTTCC,  Rev: TATGAATTCTCATTCGGAAAAGCGGGG |
| XdpB∆5 CHis | pET26b | Fwd: GATTTCATATGACCAAGACCACACTCTTCC,  Rev: TATGAATTCTCATTCGGAAAAGCGGGG |
| XdpBWT C240S | pET26b | Fwd: CAACGGTATTTCCTCCAGTGATCCCCAGAC  Rev: GTCTGGGGATCACTGGAGGAAATACCGTTG |
| XdpB∆5 C240S | pET26b | Fwd: CAACGGTATTTCCTCCAGTGATCCCCAGAC  Rev: GTCTGGGGATCACTGGAGGAAATACCGTTG |
| XdpA WT | pET26b | Fwd: TTTCATATGACTCAGATTCCCTTAGTCTTG,  Rev: AAATGCTCGAGTCACGCCGGCGTCCGATAGCGTT |
| XdpA NHis | pET28b | Fwd: TTTCATATGACTCAGATTCCCTTAGTCTTG,  Rev: AAATGCTCGAGTCACGCCGGCGTCCGATAGCGTT |
| XdpA CHis | pET26b | Fwd: TTTCATATGACTCAGATTCCCTTAGTCTTG,  Rev: AAATGCTCGAGCGCCGGCGTCCGATAGCGTT |
| XdpR WT | pET26b | fwd: GACTACATATGAAAGTCAGTCGAGAACAGATGGC,  Rev: AAATCCGAATTCCTACTCCACCTGCGTGTCGATCC |
| XdpR NHis | pET28b | fwd: GACTACATATGAAAGTCAGTCGAGAACAGATGGC,  Rev: AAATCCGAATTCCTACTCCACCTGCGTGTCGATCC |
| XdpR CHis | pET26b | fwd: GACTACATATGAAAGTCAGTCGAGAACAGATGGC,  Rev: AAATCCGAATTCCTCCACCTGCGTGTCGATCC |

**Table B. Expression system: reverse transcriptase qPCR Primers.**

| Function | Name | Orientation | 5′- .... - 3′ Sequence | Amplicon (bp) | GenBank/UniProt number |
| --- | --- | --- | --- | --- | --- |
| reference genes | *atp*D | F | AGCTACCCCAAAGAAAACCG | 118 | KXG85648.1 |
|  |  | R | TGTCAGTCTCAATCGCGTTG |  |  |
|  | *recA* | F | GGCTCGCTCAGCCTTGATG | 84 | KXG86763.1 |
|  |  | R | CGGAGCTTTCCGGTCCATA |  |  |
|  | *coxA* | F | CGACCGTTCTCTGCATGACA | 63 | KXG87943.1 |
|  |  | R | CCCAGCGACAGCACGTAA |  |  |
|  | *pro*C | F | GACCGAAGCACCGAAGAATG | 77 | KXG85914.1 |
|  |  | R | AGGACCGCATCCATCAACTGA |  |  |
|  | *rpoD* | F | TCGCATCTGGGTGACTTCATC | 73 | KXG85954.1 |
|  |  | R | CGCAGGTTGGCCTGAATGG |  |  |
| oxidative stress markers | *sodBI* | F | CAGGCATTCGCGTCTGATCTC | 86 | KXG88030.1 |
|  |  | R | CCAGCCGGAACCAAACTGT |  |  |
|  | *katG* | F | CGATGGCTTCCGCAACTTC | 97 | KXG85283.1 |
|  |  | R | GCCCTGTCAGCGTGAGAA |  |  |
|  | trxA | F | CCGTGAAAGTCGATACGTCAAAC | 87 | KXG85552.1 |
|  |  | R | CCACACCATTCAGCCCAGAA |  |  |
|  | *gshB* | F | CGCGATGACCGCAACTTC | 91 | KXG84280.1 |
|  |  | R | CGAACGTCGGGCAGATACTG |  |  |
| codeine metabolism | *xdpB* | F | CCAGACGCAGTACGATTACATTG | 85 | A0A0A0VDJ9 |
|  |  | R | GTCCGCCGGTAGCACCTT |  |  |
|  | *xdpR* | F | CAACGTCTGCGCGACAAGA | 75 | A0A0A0VAX9 |
|  |  | R | GAAGGCCGCCGGTATGAC |  |  |

**Table C. Sequential differences between the C terminal regions of the OYE C1 subgroup.**

| **OYE member belonging to *xdo* operon** | | | **OYE member is NOT a part of *xdo* operon** | | |
| --- | --- | --- | --- | --- | --- |
| **GenBank ID** | **C terminal amino acids (following GxxDYP motif)** | **Source organism** | **GenBank ID** | **C terminal amino acids (following GxxDYP motif)** | **Source organism** |
| KM272590.1 | RFSETTSDN | *Agrobacterium bohemicum* R89-1 | AFY86136.1 | SLELQAAG | *Chroococcidiopsis thermalis* PCC 7203 |
| ACK46674.1 | TLADANA | *Shewanella baltica* OS223 | Q0G6F0 | FLSEEEKTRFADAA | *Fulvimarina pelagi* HTCC2506 |
| AEX51564.1 | SLTETSK | *Rahnella aquatilis*ATCC 33071 | BAF87064.1 | TLGTAA | *Azorhizobium caulinodans* ORS 571 |
| AIU90296.1 | SLTETKK | *Pectobacterium carotovorum* subsp. *odoriferum* | AFI84958.1 | FLEPI | *Methylophaga nitratireducenticrescens* |
| ANJ99371.1 | PLSASGK | *Serratia plymuthica* | ACF52127.1 | VLEESVAA | *Stenotrophomonas maltophilia* R551-3 |
| SDU81739.1 | TIAG | *Pseudomonas corrugata* | AOS15433.1 | ALPE | *Xanthomonas oryzae* pv. *oryzae* |
| ACF51710.1 | ALTDTAAQ | *Stenotrophomonas maltophilia* R551-3 | ABE50097.1 | TLQG | *Methylobacillus flagellatus* KT |
| AKF37139.1 | PLTDTNK | *Yersinia enterocolitica* | ACZ76285.1 | FLVNSDI | *Dickeya zeae* Ech586 |
| AAK90820.1 | RFNEASGS | *Agrobacterium fabrum* str. C58 (plasmid) | A0A0C1UG09 | AI | *Hassallia byssoidea* VB512170 |
| AOO82630.1 | AIAAASRS | *Bosea vaviloviae* | ABS66576.1 | TLADAAA | *Xanthobacter autotrophicus* Py2 |
| SBW78237.1 | TLAESSTRAN | *Pseudomonas veronii* 1YdBTEX2 | AFY34059.1 | FLT | *Calothrix* sp. PCC 7507 |
| *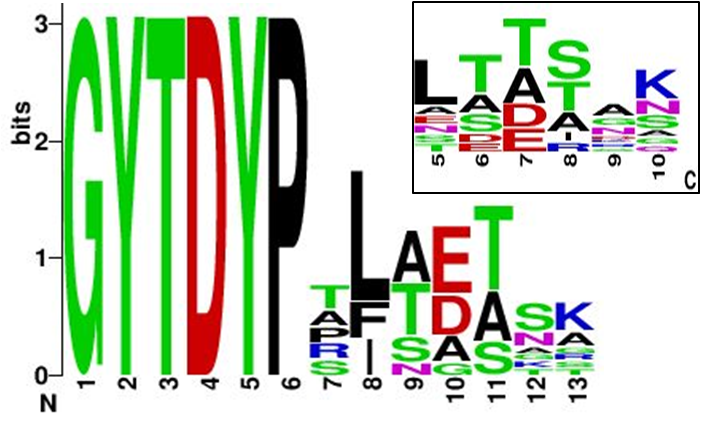* | | | *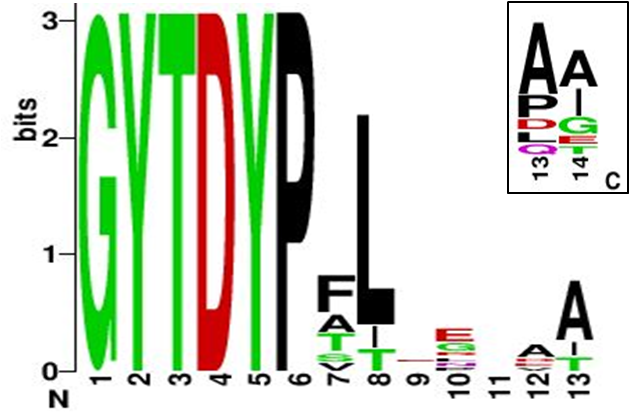* | | |

Sequence logo representation computed by WebLogo software [S5] schematically shows predominant amino acids at the end of OYE C1 proteins. Alignments were performed with respect to the conserved GxxDYP motif (large logo) or strictly starting from the C terminus (inset boxes).


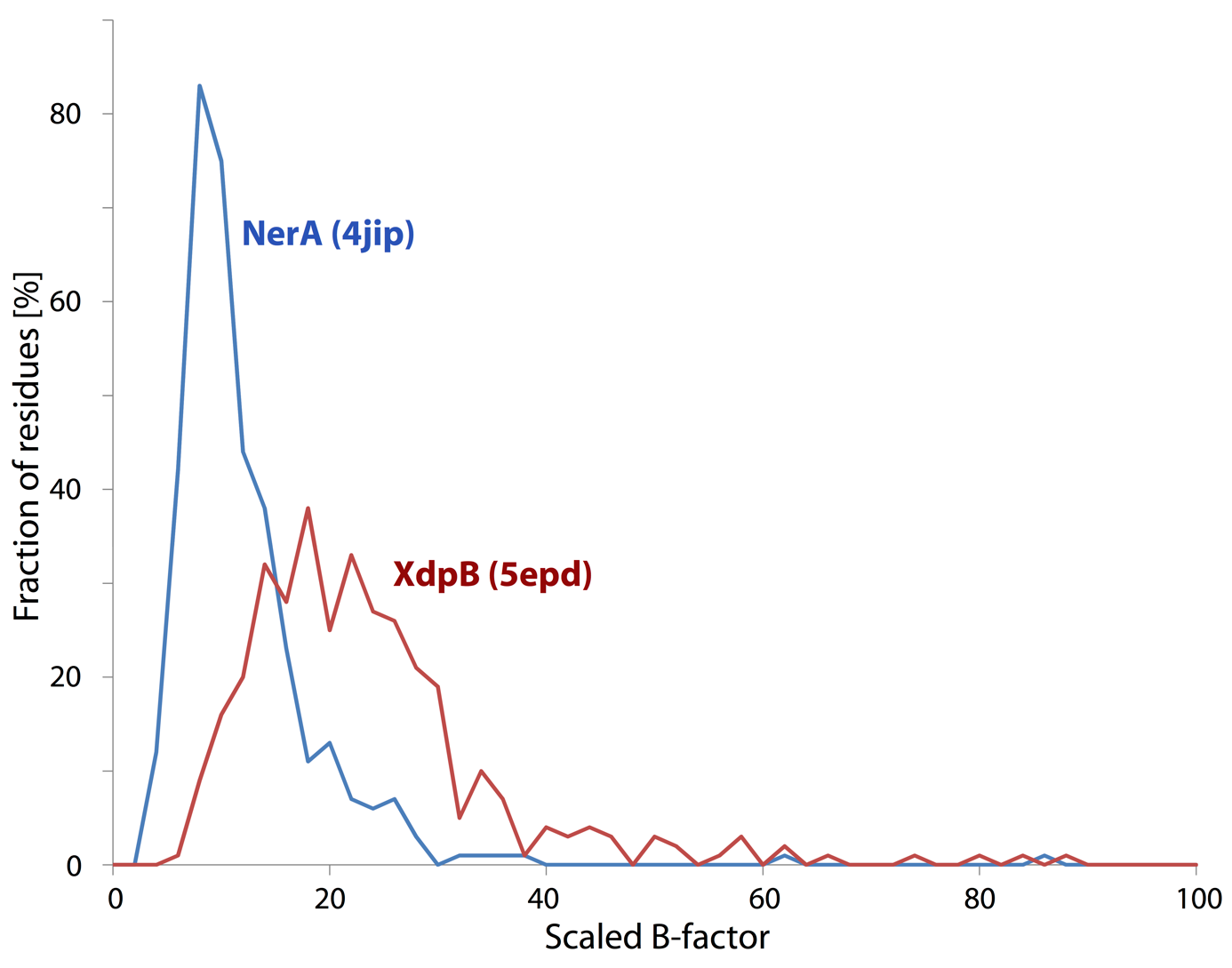


**Figure A. Histogram of the scaled B-factors.** Histogram of the scaled B-factors of NerA (PDB code 4jip, in blue) and XdpB (PDB 5epd, in red) showing that NerA has a larger fraction of residues with low values of the scaled B factors than XdpB and therefore a lower flexibility. The B values were linearly scaled for each structure individually between 1 and 100 as described in paper [S6].

**
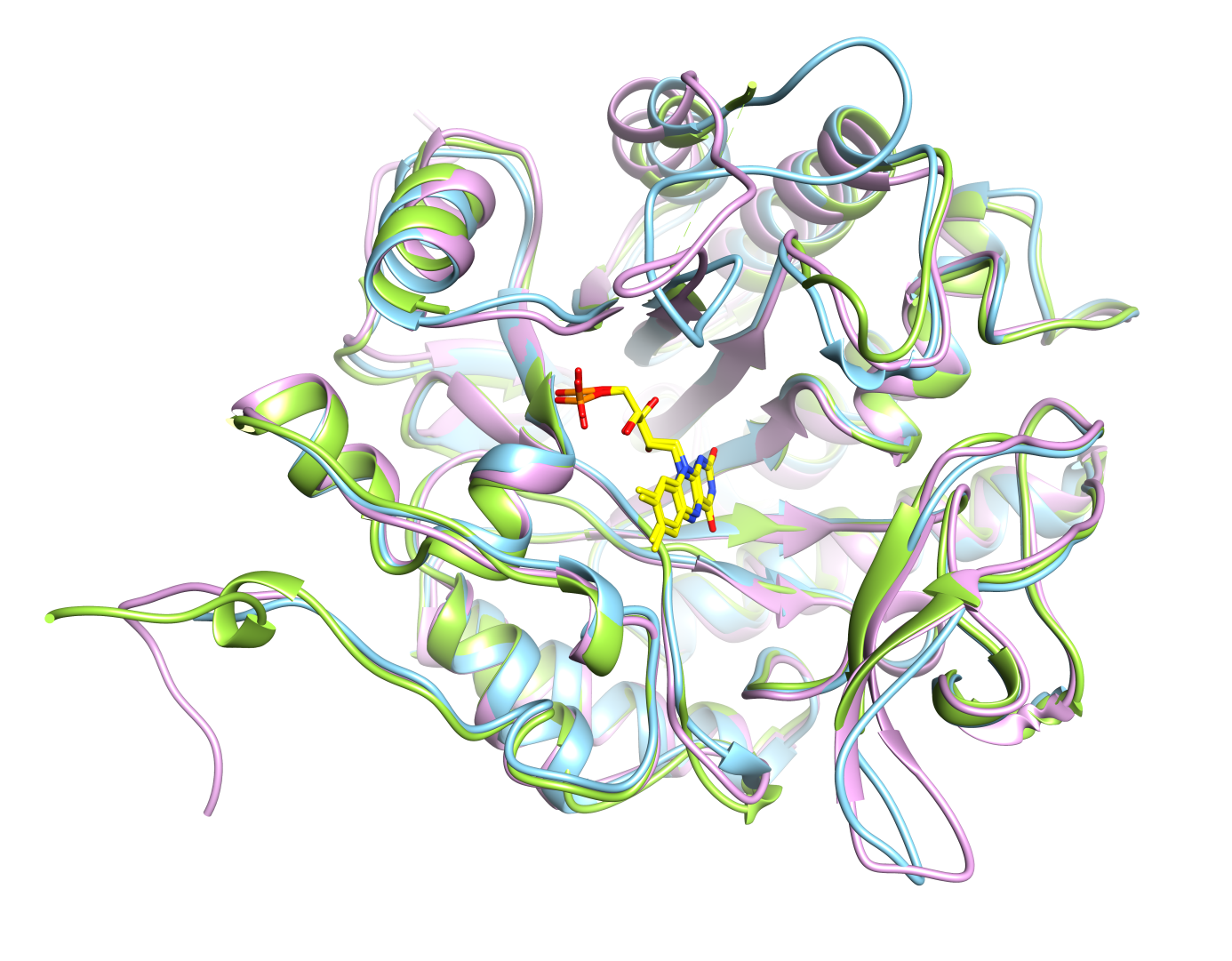
**

**Figure B. The superposition of XdpB (5epd, green), NerA (4jic, blue) and MorB (1gwj, pink).**


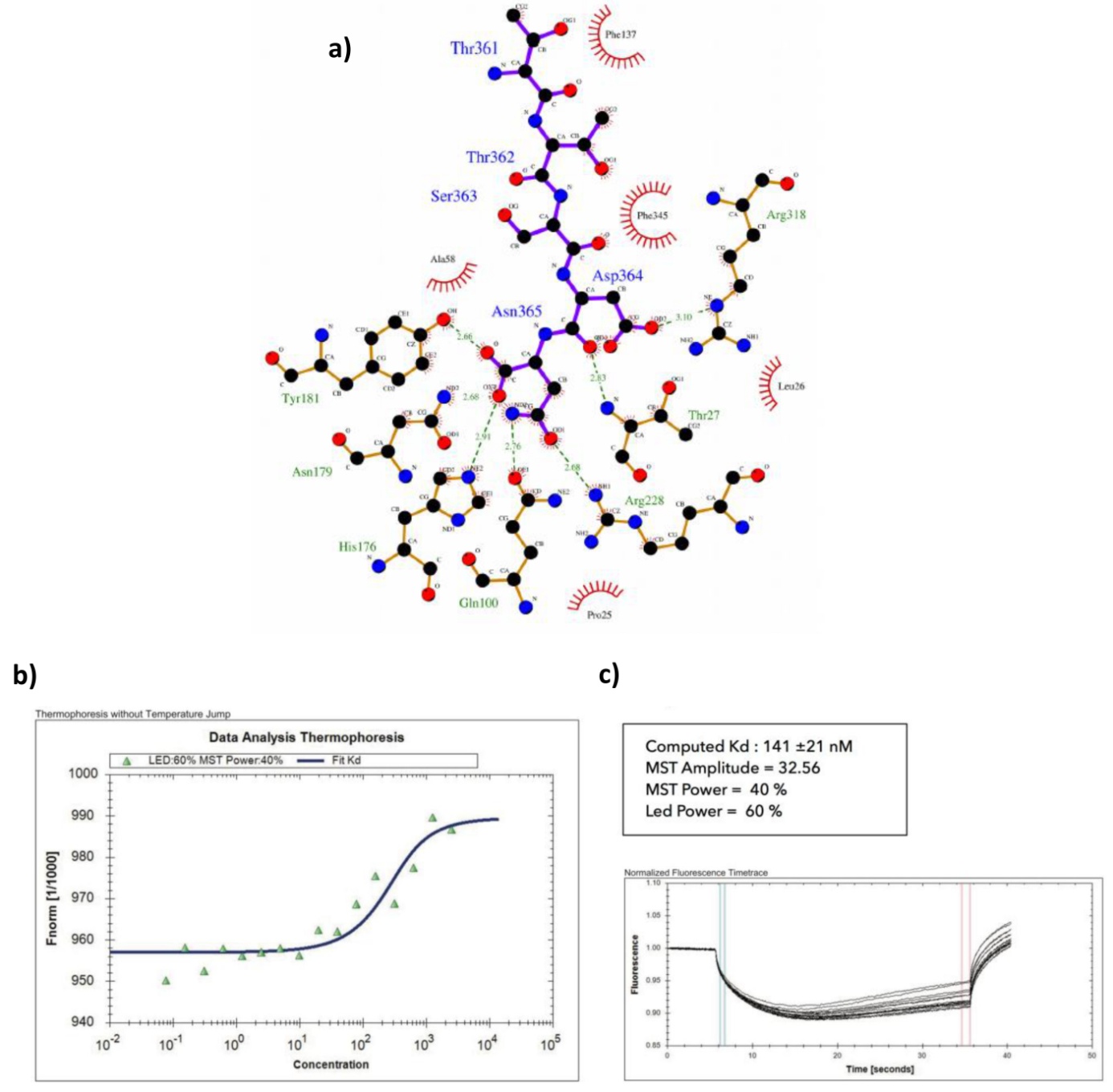


**Figure C. The binding of the C-terminal pentapeptide TTSDN to the FMN binding site of the symmetry-related in the XdpB structure.** a) Analysis of interaction drawn by LigPlot+ [S7]. b) The binding of pentapeptide TTSDN to the apo form of XdpBΔ5 (XdpB with the C-terminal TTSDN removed). The points show MST measured changes of the fluorescence signal. c) The raw MST data interpreted in b).


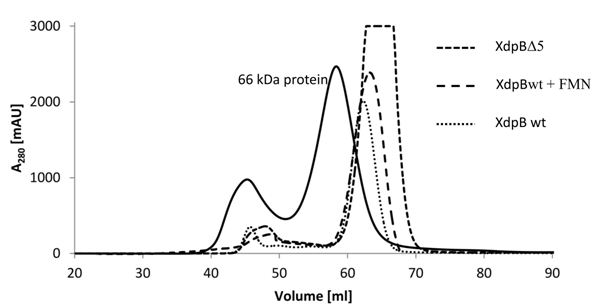


**Figure D. Elution volumes of XdpBwt, XdpBwt + 5 mM FMN, and XdpBΔ5 variant.**


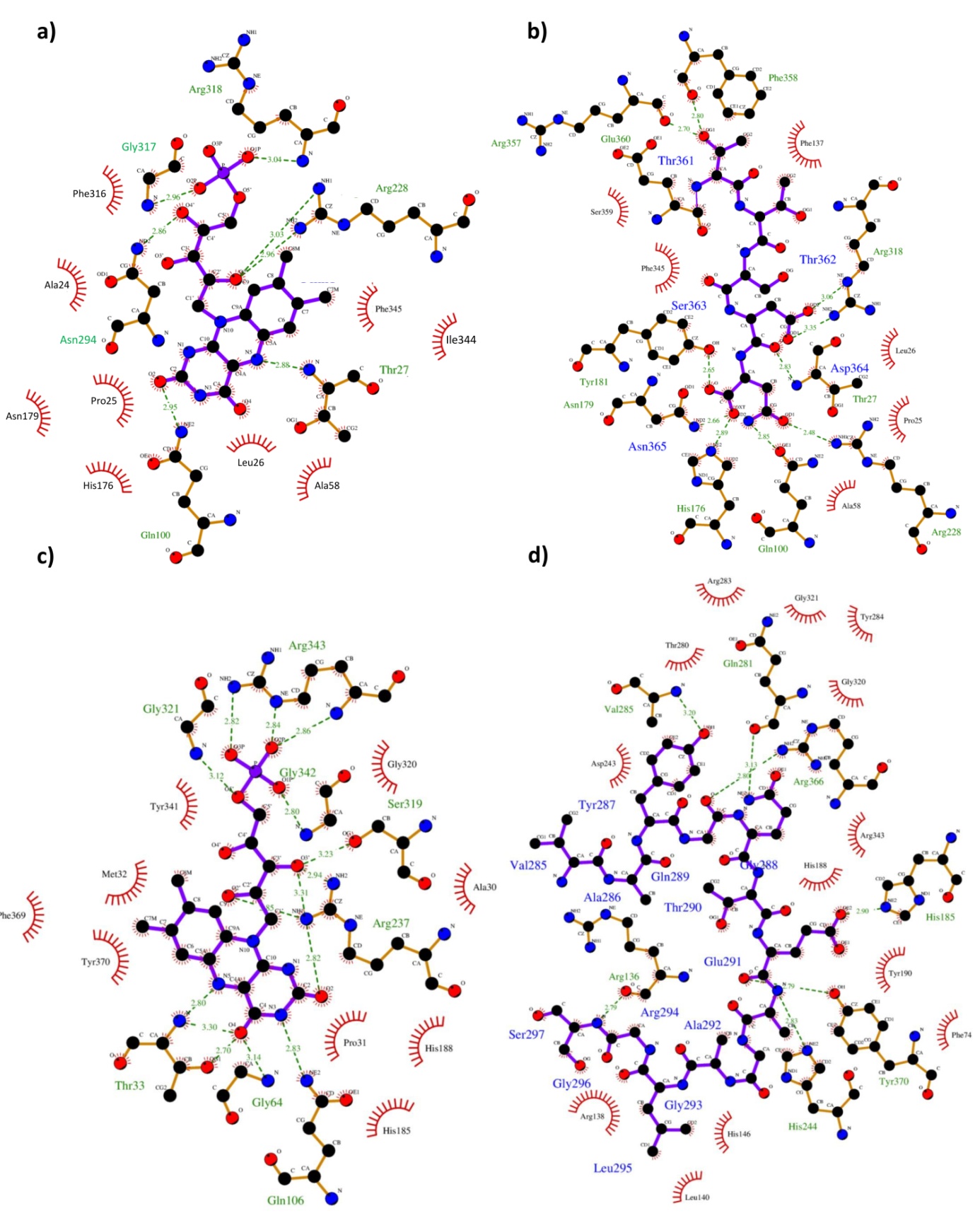


**Figure E.** **The interaction analysis of the XdpB (5epd) and OPR3 (2hsa) proteins drown by LigPlot+ software [S7].** a) Interaction in the XdpB FMN binding site predicted by docking of FMN. b) Interaction of the TTSDN peptide as observed in XdpB docking computations. c) Interaction in the OPR3 protein crystal structure of FMN (c) and interaction of one autogenic blocking loop observed in the 2hsa structure (d).

a)


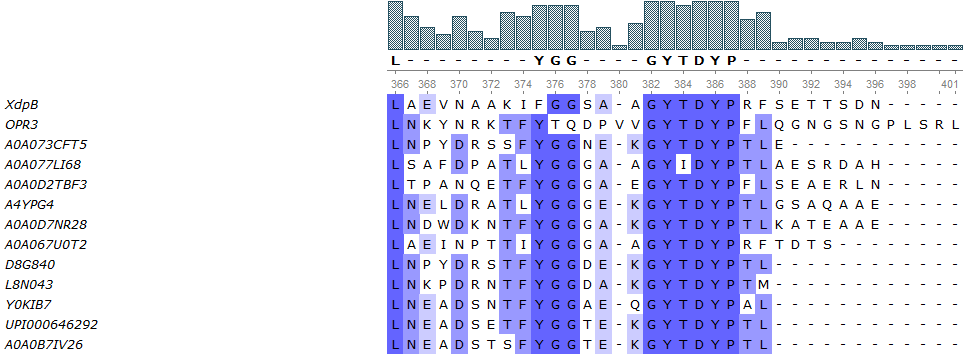


b)


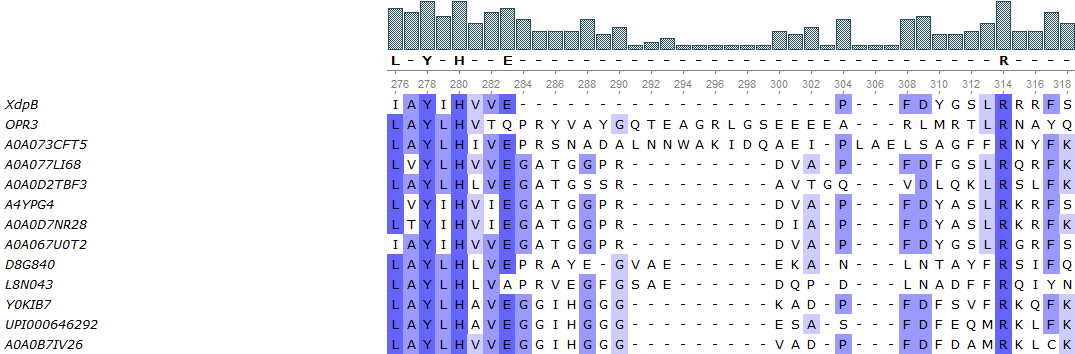


**Figure F**. **Multiple sequence alignment of representatives of OYE C1 family calculated by the ClustalW algorithm in the Ugene program [S8].** a) The C terminal region. b) The alignment shows the OPR3-like insertions [S9]. Consensus scoring is shown above the sequences. In the alignment picture, only 11 representative sequences (Uniprot codes), OPR3 and XdpB are shown. Residues highlighted in blue represent conserved amino acids.


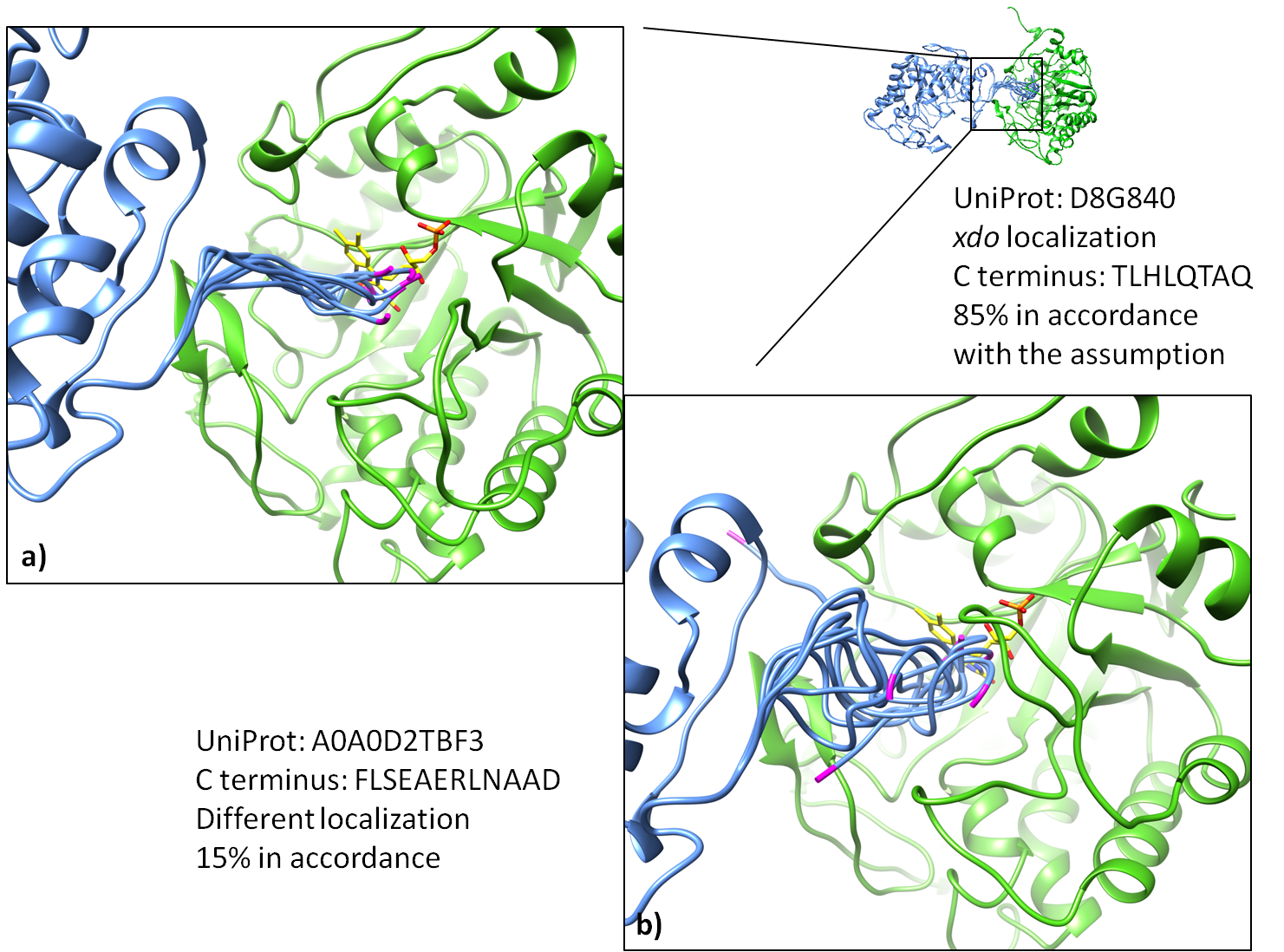


**Figure G. Computer docking of the C-termini of ten selected OYE C1 proteins (in blue and green) into the FMN binding site**. The last amino acid of each protein is highlighted in magenta. Selected proteins show significant differences: C terminus TLHLQTAQ of the subgroup C1 (on top) is more prone to interact with FMN binding site than C-termini of the other C1 subgroup OYEs. Therefore, the C terminal amino acids of OYE C1 encoded in *xdo* operon are more likely to exhibit autogenic blocking than the other OYE C1.


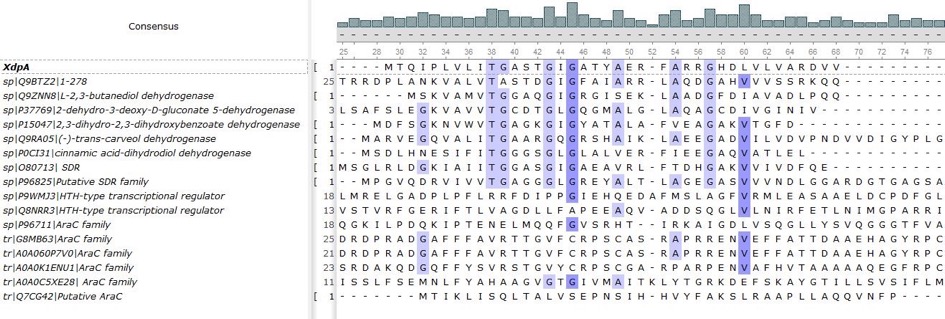


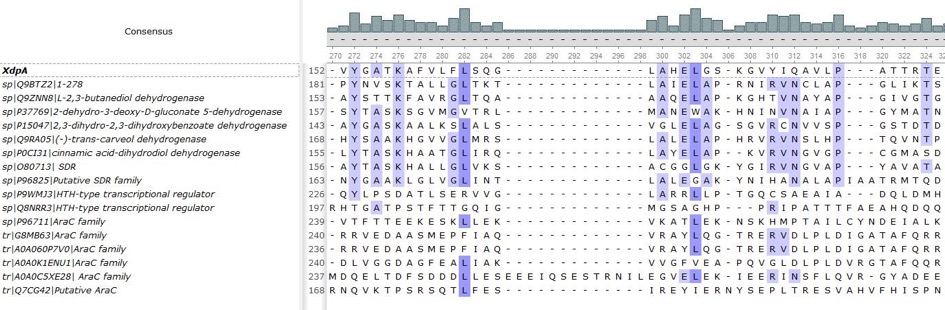


**Figure H. Multiple sequence alignment calculated in Ugene program by the ClustalW algorithm [S8].** It compares sequences of XdpA and members of the SDR family (XdpA and the first 8 representative sequences), and the AraC family (8 sequences). Consensus scoring is shown above the sequence with conserved residues in blue. XdpA bears conserved residues typical for SDR dehydrogenases (mainly in light blue) and not for AraC transcriptional regulators. Sequences are identified by Uniprot codes and annotation in left.


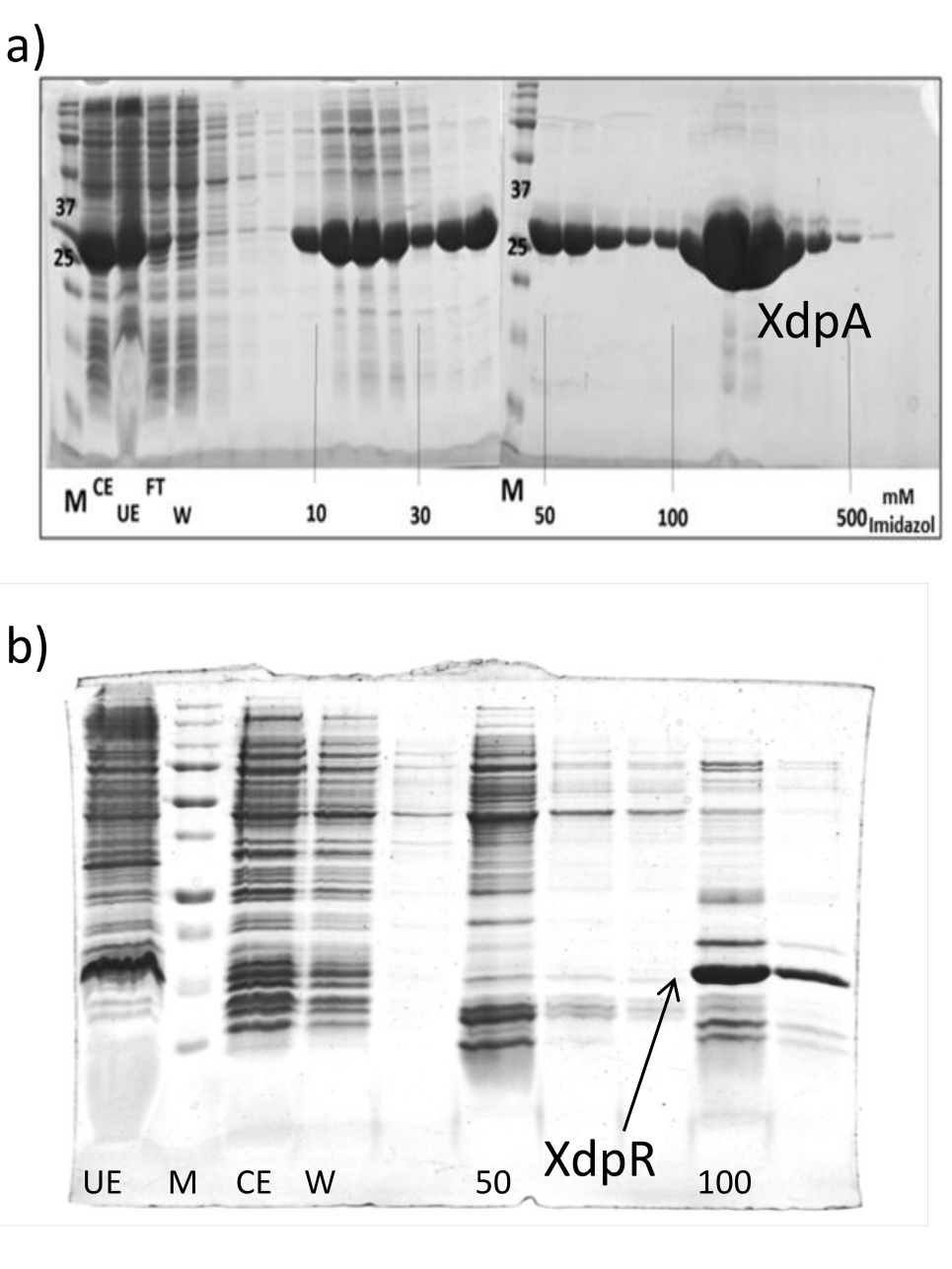


**Figure I**. P**urification of XdpA and XdpR.** a) Electrophoretogram from XdpA NiNTA agarose purification. M-molecular marker (same as in Fig.2), CE: cytosolic extract, UE: insoluble (urea) extract, FT: non-bound fraction, W: wash fractions. The numbers refer to imidazole step elution. b) Electrophoretogram from XdpR NiNTA agarose purification. Lanes same as above.

**Figure J**. **Relative quantification of gene expression by RT qPCR.** Comparison of *xdpB* expression and selected oxidative stress markers of strain R89-1 grown on LBTE medium (1) and on LBTE supplemented with 1g/L codeine (2). The sample grown in LB medium (13.5 h) was used as a reference and the *coxA* gene served as an endogenous control. All experiments were done in independent triplicates.

**References**

[S1] Reinhard L, Mayerhofer H, Geerlof A, Mueller-Dieckmann J, Weiss MS. Optimization of protein buffer cocktails using Thermofluor. Acta Cryst. F. 2013;69(Pt 2):209-214.

[S2] Forneris F, Orru R, Bonivento D, Chiarelli LR, Mattevi A. ThermoFAD, a Thermofluor®-adapted flavin ad hoc detection system for protein folding and ligand binding. FEBS Journal. 2009;276(10):2833-2840.

[S3] Webb B, Sali A. Comparative Protein Structure Modeling Using Modeller. Current Protocols in Bioinformatics. 2014; John Wiley & Sons, Inc.;5.6.1-5.6.32

[S4] Edgar RC. MUSCLE: multiple sequence alignment with high accuracy and high throughput. Nucleic Acid Research. 2004;32(5):1792-1797.

[S5] Crooks GE, Hon G, Chandonia JM, Brenner SE. WebLogo: A sequence logo generator, Genome Research. 2044;14:1188-1190.

[S6] Schneider B, Gelly J-C, de Brevern AG, Černý J. Local dynamics of proteins and DNA evaluated from crystallographic B factors. Acta Crystallographica Section D: Biological Crystallography. 2014;70(Pt 9):2413-9.

[S7] Laskowski RA, Swindells MB. LigPlot+: multiple ligand-protein interaction diagrams for drug discovery. Journal of Chemical Information and Modeling 2011;**51**(10):2778-2786.

[S8] Okonechnikov K, Golosova O, Fursov M, team tU. Unipro UGENE: a unified bioinformatics toolkit. Bioinformatics. 2012;28(8):1166-7.

[S9] Breithaupt C, Kurzbauer R, Lilie H, Schaller A, Strassner J, Huber R, et al. Crystal structure of 12-oxophytodienoate reductase 3 from tomato: Self-inhibition by dimerization. Proceedings of the National Academy of Sciences of the United States of America. 2006;103(39):14337-42.
